# One step fabrication of aligned carbon nanotubes using gas rectifier

**DOI:** 10.1038/s41598-022-05297-6

**Published:** 2022-01-25

**Authors:** Toshihiko Fujimori, Daiji Yamashita, Yoshiya Kishibe, Momoko Sakai, Hirotaka Inoue, Takamasa Onoki, Jun Otsuka, Daisuke Tanioka, Takeshi Hikata, Soichiro Okubo, Keishi Akada, Jun-ichi Fujita

**Affiliations:** 1 Sumitomo Electric Industries, Ltd., 1-1-3 Shimaya, Konohana-ku, Osaka, 554-0024 Japan; 2 Institute of Applied Physics, University of Tsukuba, Tsukuba, 305-8573 Japan

**Keywords:** Carbon nanotubes and fullerenes, Chemical engineering

## Abstract

We report the one-step fabrication of aligned and high-quality carbon nanotubes (CNTs) using floating-catalyst chemical vapor deposition (FCCVD) with controlled fluidic properties assisted by a gas rectifier. The gas rectifier consists of one-dimensional straight channels for regulating the Reynolds number of the reaction gas. Our computational fluid dynamics simulation reveals that the narrow channels of the gas rectifier provide steady and accelerated laminar flow of the reaction gas. In addition, strong shear stress is induced near the side wall of the channels, resulting in the spontaneous formation of macroscopic CNT bundles aligned along the direction of the gas flow. After a wet-process using chlorosulfonic acid, the inter-tube voids inherently observed in as-grown CNT bundles are reduced from 16 to 0.3%. The resulting CNT fiber exhibits a tensile strength of 2.1 ± 0.1 N tex^−1^ with a Young’s modulus of 39 ± 4 N tex^−1^ and an elongation of 6.3 ± 0.6%. FCCVD coupled with the strong shear stress of the reaction gas is an important pre-processing route for the fabrication of high-performance CNT fibers.

## Introduction

There is an ever-increasing need for robust lightweight materials in a variety of fields on automotive, architectural, and aerospace applications^1,2^. To this end, carbon nanotubes (CNTs), which possess unique cylindrical nanostructures connected with *sp*^2^ C–C bonding, are promising candidates owing to their outstanding electrical^3^, mechanical^4,5^, and thermal^6^ properties at the nanoscale. However, their macroscopic counterparts (e.g., assembly into fibers) demonstrate the properties far below than those expected for individual CNTs mainly due to the misorientation and low packing density of individual CNTs and/or CNT bundles, as well as the presence of defects and short length (typically on the order of micrometers)^7,8^. The structural entanglement incorporated in CNT fibers originates from the experimental difficulty in controlling the parallel alignment and hexagonal stacking of CNTs during the synthesis process. Therefore, a major challenge in developing macroscopic CNT fibers is currently focused on the use of post-processing techniques (dry spinning^9–13^, wet spinning^14–16^, combined dry–wet spinning^17^, microcombing^18^, and chemical crosslinking^19^) to achieve uniaxial alignment and packing of CNTs.

Despite extensive research on post-processing techniques, little is known about pre-processing techniques such as a direct route for the simultaneous production and alignment of CNT fibers. Among the various synthesis methods of CNTs^20–23^, floating-catalyst chemical vapor deposition (FCCVD) enables the continuous and scalable production of a CNT aerogel (also referred to as an elastic smoke of CNTs)^10,24^. To control the growth direction of CNTs, fluidics-based engineering plays an important role, such as in the growth of a straight CNT under laminar flow with a low Reynolds number and the growth of a wavy CNT under turbulent flow with a high Reynolds number^25,26^. However the flow-directed growth of CNTs, known as the “kite-mechanism”, has been achieved using a flat surface with immobilized catalytic nanoparticles, maintaining large inter-tube distances to prevent entanglement through van der Waals attraction^27^. Accordingly, a key challenge is to achieve flow-directed growth and alignment of CNTs simultaneously, in conjunction with use of the FCCVD method by controlling the fluid properties of the reaction gases.

Here we report an FCCVD method for the one-step fabrication of aligned CNT bundles using a gas rectifier (so-called honeycomb ceramic filters), which possesses one-dimensional straight channels for regulating the Reynolds number of a gaseous medium containing a growing CNT aerogel. In addition to the gas rectification effect, we found that a sterically constrained space inside the channel induced accelerated laminar flow with strong shear stress near the channel walls, resulting in the spontaneous condensation of floating CNTs along the direction of gas flow. Although inter-bundle voids remain in the as-grown sample, the CNT bundles can be densified into the fibers with improved alignment by further wet-stretching process using chlorosulfonic acid (CSA), which is a good solvent for the dispersion of CNTs without the aid of dispersants^14^.

## Materials and methods

Aligned CNT bundles were fabricated using a laboratory-designed horizontal gas-flow reactor consisting of an alumina tube (inner diameter: 42 mm, length: 1600 mm) with a gas rectifier made of alumina (square-shaped pore, pore size: 1.6 mm × 1.6 mm, cell density: 100 cpsi). The gas rectifier was set at the reactor exit. The detailed setup and experimental conditions of the reactor are shown in Supplementary Fig. S1. We used Ar (99.9999%) as the purging gas, H_2_ (99.99999%) as the carrier gas, and C_2_H_4_ (99.9%) as a carbon source. The reactor was heated to synthesis temperature at 1473–1673 K under Ar gas flow (2 L min^−1^). H_2_ was then injected (1.3 L min^−1^) and Ar gas flow was stopped. Subsequently, H_2_ (10 L min^−1^), C_2_H_4_ (50 mL min^−1^), and a precursor solution were injected through a spray nozzle. The precursor solution was prepared by dissolving 4 wt% ferrocene (purity: 98%, Sigma-Aldrich) as the catalytic source and 2 wt% thiophene (purity: 97.0%, FUJIFILM Wako Pure Chemical Corporation) as the promoter, in toluene (purity 99.5%, FUJIFILM Wako Pure Chemical Corporation). Both C_2_H_4_ and toluene act as the hybrid carbon source^21^. During the synthesis process, the precursor solution was injected at a flow rate of 100 μL min^−1^. In the FCCVD process using the hybrid carbon source, the atomic ratios of Fe:C, S:C, and Fe:S were 0.0017, 0.0019, and 0.90, respectively (Supplementary Table S1). The as-grown CNT bundles synthesized at 1673 K were used in subsequent experiments unless otherwise specified. We also produced CNTs at the same synthesis temperature and flow rates without using the gas rectifier for comparison. To visualize the growth process of CNTs, a transparent quartz tube (inner diameter: 46 mm, length: 1600 mm) was used at a synthesis temperature of 1473 K.

The individual as-grown CNT bundles were collected as thick threads, which were then cut into short threads with a typical length of 5 cm (Supplementary Fig. S2a). To densify the as-grown CNT bundles using a wet-stretching process^28^, the CNT threads were immersed in CSA (purity: 97%, FUJIFILM Wako Pure Chemical Corporation) for 1 min. During this process, the fibrous CNTs were strained by tweezers several times. The strained samples were then pulled out from the CSA and immediately immersed in chloroform to coagulate, thus form a fiber. This process was repeated 10 times. The CNT fibers were washed with fresh chloroform to remove the remaining CSA on the fibers, and subsequently dried at 373 K under vacuum for 1 h. The resulting CNT fibers were stretched to approximately 270% compared to the initial length (Supplementary Fig. S2b). The wet-stretching process was performed inside a glove box under an Ar atmosphere. For comparison, an as-grown sample was immersed in chloroform for 10 min, forming a fiber through liquid condensation (denoted as “as-made CNT fiber”) (Supplementary Fig. S3), which was then used for mechanical testing.

Thermogravimetric analysis (TGA) was performed under N_2_/O_2_ (8:2) gas flow (200 mL min^−1^) in the temperature range of 300–1200 K at a scan rate of 3 K min^−1^ (Thermo Plus EVO2, Rigaku). The morphology of the CNT samples was observed using a scanning electron microscope (SEM; JSM-7200F, JEOL) operated at 15 kV. To evaluate the two-dimensional Chebyshev orientation parameters of the specimens, Fourier transform image analysis technique was performed using the FibreCOP software^29,30^. The CNT samples were cut using a focus ion beam SEM system (Helios NanoLab 600i, FEI) operated at 30 kV. The porosity of the CNT samples was evaluated by analyzing cross-sectional SEM images using ImageJ 1.52i software (https://imagej.nih.gov/ij/)^31^. Raman spectra were obtained with the 532 nm laser excitation using a single-monochromator Raman spectrometer (in-Via™ Raman microscope, Renishaw). To obtain polarized Raman spectra, half-wave plates and a polarizer were used to rotate the polarization of the incident and/or scattered light. The linear density of the CNT fibers was determined gravimetrically using a microbalance with a precision level of five decimals. Multiple fibers with a known total length (typically ~ 1 m) were used to measure the weight of the specimens. Mechanical testing was performed using a vertical-type force/displacement tester (FSA-1KE-5N, Imada Co., Ltd.) equipped with a force gauge (ZTA-5N, Imada Co., Ltd.) and a motorized test stand (EMX-1000N, Imada Co. Ltd.). The maximum load of the force gauge was 5 N with a precision of 1 mN. The specimen was pulled automatically using a 0.5 mm min^−1^ extension rate. The gauge length of each specimen was 10 mm.

A computational fluid dynamics (CFD) simulation was performed with a steady-state and compressible flow model using the OpenFOAM software v2012 (https://www.openfoam.com/)^32^ to simulate the distributions of gas velocity and temperature inside the model structure consisting of a gas rectifier and a cylindrical tube. The SIMPLE algorithm implemented in OpenFOAM software was used to obtain the numerical solutions. The continuity, momentum, and energy equations are respectively

1 $$\nabla \cdot \left(\rho {\varvec{u}}\right)=0,$$

2 $$\rho {\varvec{u}}\cdot \nabla {\varvec{u}}=-\nabla p+\nabla \cdot \left[\mu \left\{\nabla {\varvec{u}}+{\left(\nabla {\varvec{u}}\right)}^{T}-\frac{2}{3}\nabla \cdot {\varvec{u}}{\varvec{I}}\right\}\right]+\rho {\varvec{g}},$$

3 $$\nabla \cdot \left(\rho h{\varvec{u}}\right)+\nabla \cdot \left(\rho K{\varvec{u}}\right)=\nabla \cdot \left(\alpha \nabla h\right)+\rho {\varvec{g}}\cdot {\varvec{u}}+aG-4e{\sigma }_{\mathrm{SB}}{T}^{4},$$

where $\rho$ is the density of the gas ($\rho =pW/RT$, where *p*: the fluidic pressure, *W*: the molecular weight, *R*: the gas constant, and *T*: temperature), ${\varvec{u}}$ is the velocity field of the gas, $\mu$ is the viscosity of the gas, ${\varvec{g}}$ is the acceleration of gravity, $h$ is the specific enthalpy ($h={C}_{\mathrm{p}}\left(T-{T}_{0}\right)$, where ${C}_{\mathrm{p}}$: the specific heat capacity at constant pressure and ${T}_{0}$: standard temperature), $K$ is the kinetic energy of the gas, $\alpha =k/{C}_{\mathrm{p}}$ ($k$: the thermal conductivity of the gas), *a* is the absorption coefficient, *G* is the incident radiation intensity, *e* is the emission coefficient, and ${\sigma }_{\mathrm{SB}}$ is the Stefan-Boltzmann constant. The term $\mu$ at a given temperature was evaluated using Sutherland’s law implemented in OpenFOAM software. Details of the boundary conditions for the CFD simulation are shown in Supplementary Fig. S4. Because H_2_ was the main component of the process gas, we used the fluidic parameters of pure H_2_ gas for the CFD simulation. CFD simulation results were obtained after the residuals reached the convergence criteria (Supplementary Fig. S5). To verify the simulation model, the simulated mean velocity inside a channel of the gas rectifier was compared with the velocity calculated theoretically using the equation of state for an ideal gas. The simulated mean velocity (0.7934 m s^−1^) was in good agreement with the theoretical velocity (0.7924 m s^−1^), indicating the validation of the simulation model.

## Results and discussion

**Figure 1:**
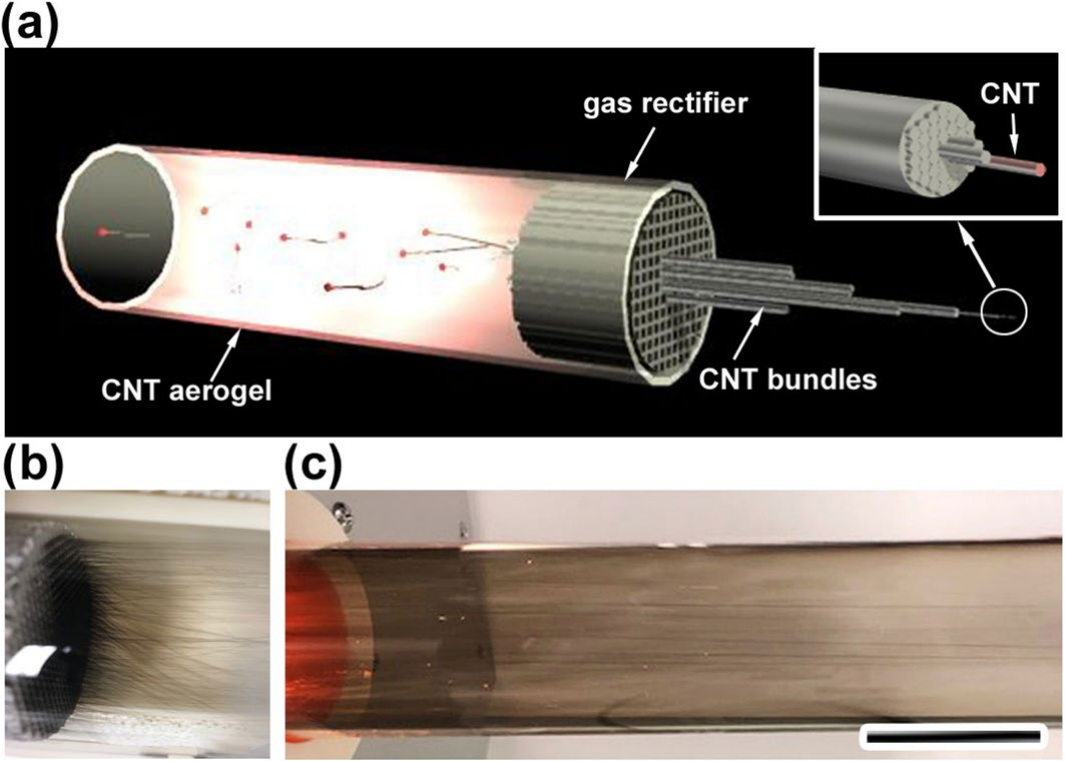
(**a**) Schematic of the fabrication process of aligned CNT bundles, demonstrating that floating CNTs flow into the straight channels of a gas rectifier, macroscopic CNT bundles then protrude from the outlet of the gas rectifier. (**b**) A photograph of the gas rectifier taken after the fabrication of CNT bundles. (**c**) CNT bundles growing in the direction of gas flow (from left to right in the photograph). Scale bar: 5 cm.

Figure 1a shows a schematic of the fabrication of CNT bundles using our FCCVD method. The formation of the CNT aerogel is believed to proceed after the decomposition of sublimated ferrocene molecules onto active iron nanoparticles and a subsequent catalytic reaction to grow CNTs on the iron nanoparticles^21,24,33^. In the conventional FCCVD regime (the synthesis process without using a gas rectifier), we observed a hollow assembly consisting of randomly oriented CNTs (hereafter denoted as “as-grown CNT web”) (Supplementary Fig. S6). In our synthesis setup, the gas rectifier was set at the reactor exit, where the temperature decreased abruptly from the synthesis temperature to approximately 1200 K. In contrast to the formation of the CNT web, we found that macroscopic CNT bundles (denoted as “as-grown CNT bundles”) protruded from the individual channels of the gas rectifier (Fig. 1b and Supplementary Movie 1) and the CNT bundles grew along the direction of the gas flow (Fig. 1c). The synthesis process can be performed until clogging of the pores occurs (< 1 h). In addition, we observed that small fragments of CNTs passed through the channels, accumulating on the mesh filter that was placed downstream of the reactor (Supplementary Fig. S1a). The fraction of as-grown CNT bundles that passed through the gas rectifier was less than 10%. The total production rate including the total amounts of CNTs (the as-grown CNT bundles attached to the gas rectifier, the CNTs that passed through the channels, and the CNTs attached to the mesh filter), other carbonaceous species, and catalysts increase from approximately 1.8 mg min^−1^ at 1473 K to 10 mg min^−1^ at 1673 K (Supplementary information S7). Under our experimental conditions, the highest production rate was obtained at a synthesis temperature at 1673 K. Here, the carbon efficiency, ${\eta }_{C}$ (%), is defined as

4 $${\eta }_{C}= {\dot{n}}_{out} / {\dot{n}}_{in},$$

where ${\dot{n}}_{out}$ and ${\dot{n}}_{in}$ are the production amount of the total graphitic carbon (mol) and the total supplied carbon (mol), respectively, in a given synthesis time^34^. We performed TGA to evaluate the ${\dot{n}}_{out}$ value at 1673 K, which reveals a graphitic carbon content of 57 wt% (Supplementary Fig. S8). Considering the graphitic carbon content, the carbon efficiency at 1673 K is 4.3%. After the synthesis, one end of the as-grown CNT bundles was attached to the end of an individual channel of the gas rectifier and the other end to the mesh filter that was placed downstream of the reactor (Supplementary Fig. S1a). To collect the sample, the as-grown CNT bundles attached to the mesh filter were cut off, and the gas rectifier with the as-grown CNT bundles was removed from the reactor tube. Finally, the as-grown CNT bundles were removed from the gas rectifier.

**Figure 2:**
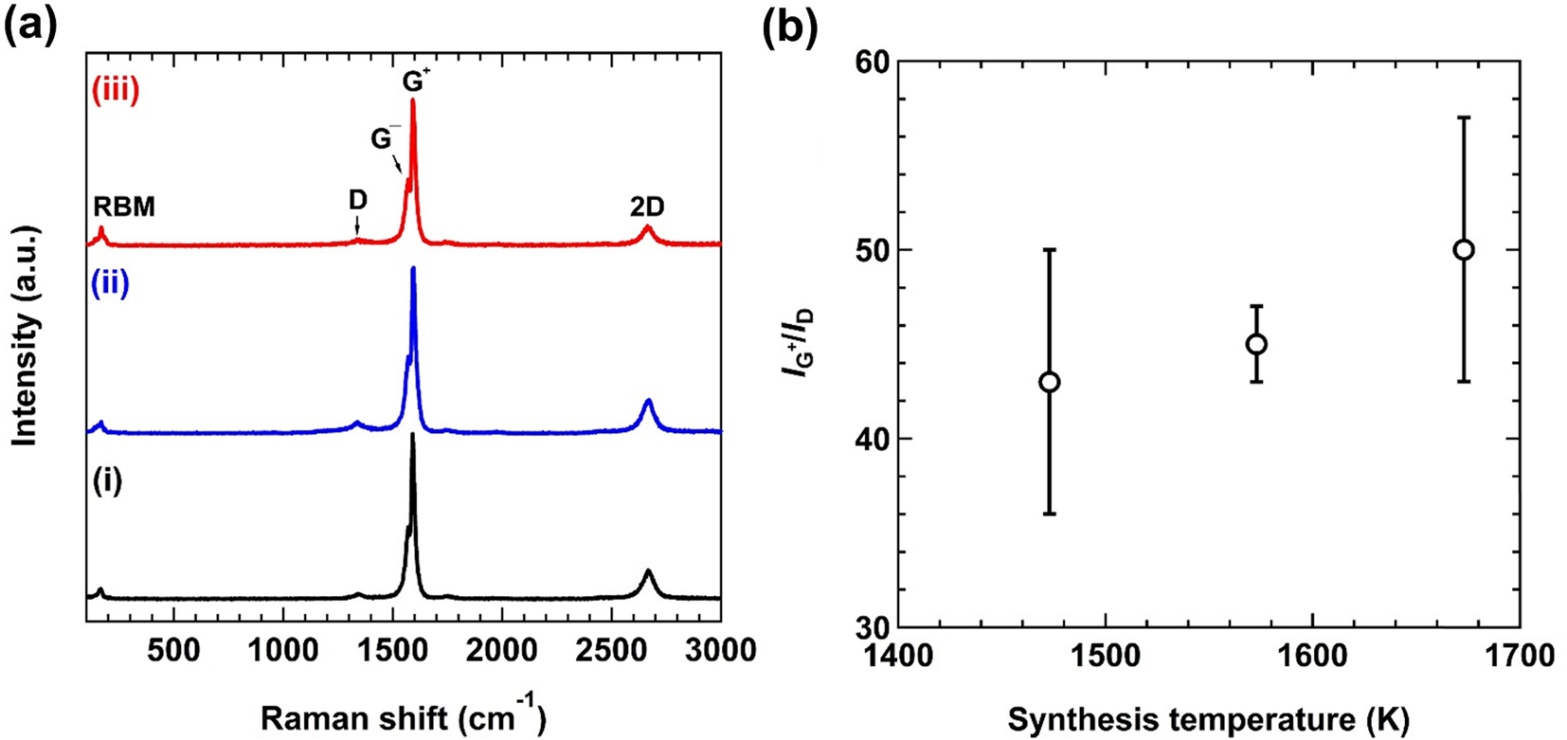
(**a**) Raman spectra of as-grown CNT bundles fabricated at the synthesis temperature of (i) 1473 K, (ii) 1573 K, and (iii) 1673 K, measured with the 532 nm laser excitation. (**b**) Synthesis temperature dependence of ${I}_{{\rm G}^{+}}/{I}_{\rm D}$ for as-grown CNT bundles evaluated from the Raman spectra shown in (**a**).

To assess the quality of as-grown CNT bundles, we performed Raman spectroscopy. Raman spectra of as-grown CNT bundles show characteristic vibrational modes of CNTs at 170, 1570, 1590, and 2672 cm^−1^, which are assigned to radial breathing mode (RBM), G^−^ peak, G^+^ peak, and 2D peak, respectively (Fig. 2a)^35^. Quantitative information of the defect density of the CNT samples was evaluated by the intensity ratio of the G^+^ mode and the defect-induced D mode (~ 1340 cm^−1^)^35^. Regardless of the synthesis temperature tested in this study, high ${I}_{{\rm G}^{+}}/{I}_{\rm D}$ values over 40 are obtained for as-grown CNT bundles and the ${I}_{{\rm G}^{+}}/{I}_{\rm D}$ values slightly increase as the synthesis temperature increases, revealing the efficient fabrication of high-quality CNTs with lower defect densities and fewer carbon impurities (Fig. 2b).

**Figure 3:**
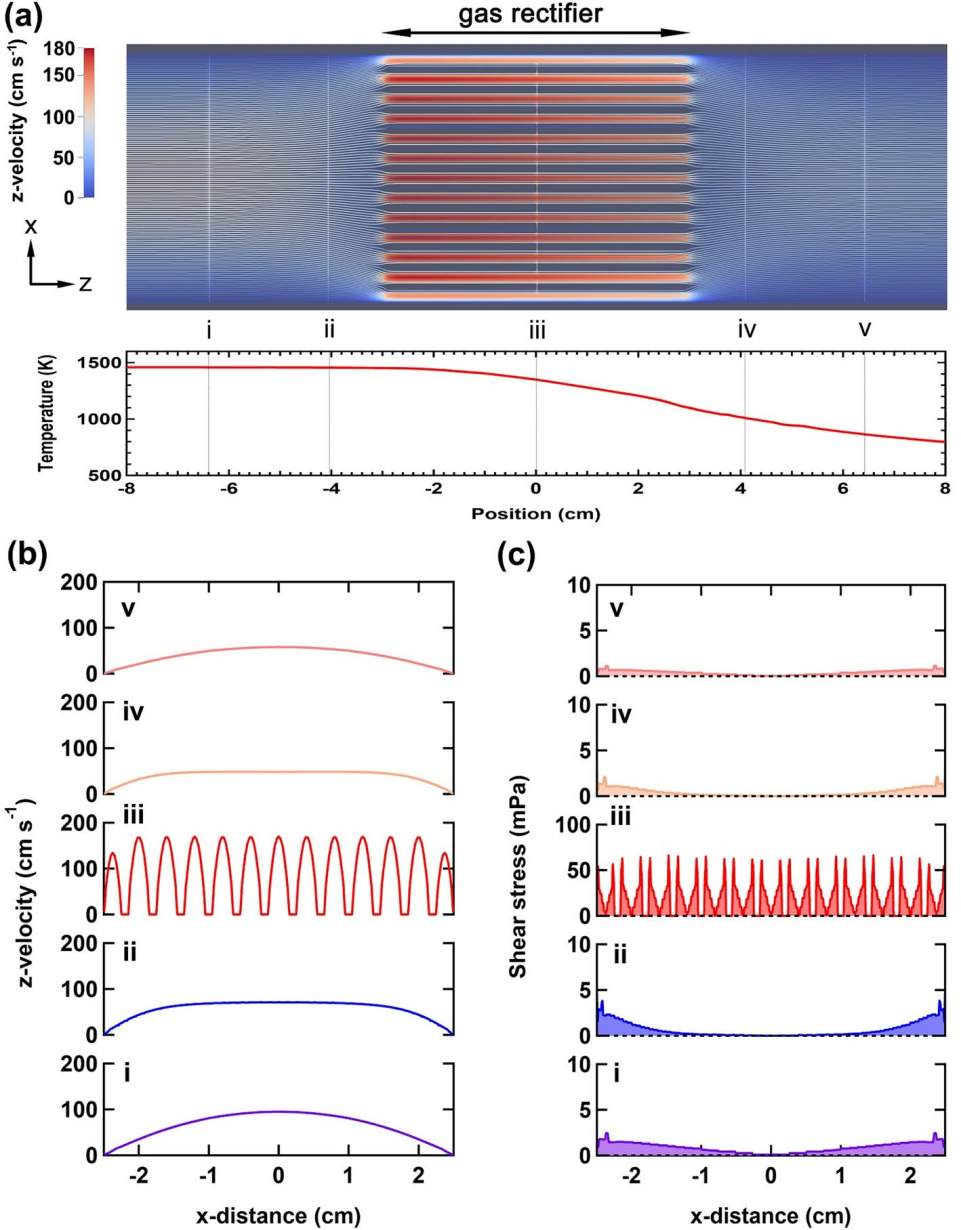
(**a**) Simulated steam lines of the gas (upper) and temperature profile (lower) near the gas rectifier. The gas flow is set from left to right. (**b**) The *z*-velocity profiles plotted along the *x* direction at position **i**–**v**. (**c**) Profiles of calculated shear stress at the position **i**–**v** of the reactor. The CFD simulation was performed with the OpenFOAM software (https://www.openfoam.com/).

To understand the effect of the gas-rectifier on the fluid properties, we performed CFD simulations (Fig. 3). Figure 3a shows a simulated *z*-velocity profile of hydrogen gas along the long axis (*z*-direction in Fig. 3a) of the horizontal reactor (upper) and the temperature profile (lower), which reproduces the experimental temperature profile near the gas rectifier. The *z*-velocity profile is parabolic at position **i**, where the temperature is identical to the synthesis temperature for growing the CNT aerogel in the gas phase (Fig. 3b). The Reynolds number in a pipe is expressed as

5 $${R}_{e}=\rho (T)u(T){D}_{\mathrm{H}}/ \mu (T),$$

6 $${D}_{\rm H}= 4\it{A}/\it{P}$$

where *ρ*(*T*) is the density, *u*(*T*) is the characteristic *z*-velocity, *μ*(*T*) is the dynamic viscosity, and *T* is the temperature of the process gas^36^. *D*_H_ is the hydraulic diameter, *A* is the cross-sectional area, and *P* is the wetted perimeter of the pipe. Considering the temperature dependence of the density and dynamic viscosity of hydrogen gas (Supplementary Fig. S9), *R*_*e*_ is estimated to be 31 at position **i**, reflecting laminar flow inside the reactor tube (the range of laminar flow is known to be *R*_*e*_ < 2000). The fluidic parameters and details for calculating *R*_*e*_ are shown in Supplementary Table S2. When passing through the narrow channels inside the gas rectifier (position **iii** in Fig. 3a), the *z*-velocity becomes 1.8 times higher than that inside the reactor tube (Fig. 3b). The Reynolds number is lower (*R*_*e*_ = 2.2) because the accelerated gas passes the restricted volume with a narrower diameter (*D*_H_ = 1.6 and 45 mm for the channel and reactor tube, respectively). The abrupt decrease in *R*_*e*_ indicates that the straight channel of the gas rectifier offers more steady and fast laminar flow, which is the preferential condition for producing a straight CNT along the direction of gas flow^25^. The gas is then exhausted from the gas rectifier with decreasing temperature, subsequently diffusing into the larger volume of the reactor tube (positions **iv–v** in Fig. 3a).

In addition to the gas rectification effect that controls the directed flow of CNTs, strong shear stress induced by the accelerated laminar flow inside the channel plays an important role in the fabrication of aligned CNT bundles. According to fluid dynamics, the shear stress, *τ* can be expressed by

7 $$\tau =\mu \left(T\right) du\left(T\right)/dx,$$

where the differential term corresponds to the gradient of the *z*-velocity profiles shown in Fig. 3b. Thus, maximum shear stress occurs as a result of the steep change in the *z*-velocity at the interface of the gas and sidewalls. The evaluated maximum shear stresses are 2.5 mPa and 62 mPa inside the reactor tube (position **i**) and in the channels of the gas rectifier (position **iii**), respectively (Fig. 3c), indicating that the narrow channels lead to the remarkable enhancement of shear stress under laminar flow. This trend is not limited to the interface, it is also observed in the intermediate space between the sidewall and the center of the channels. Although the shear stress decays abruptly and finally reaches zero at the center of the channel, the intermediate shear stress is still higher than that obtained at the interface of the reactor tube.

**Figure 4:**
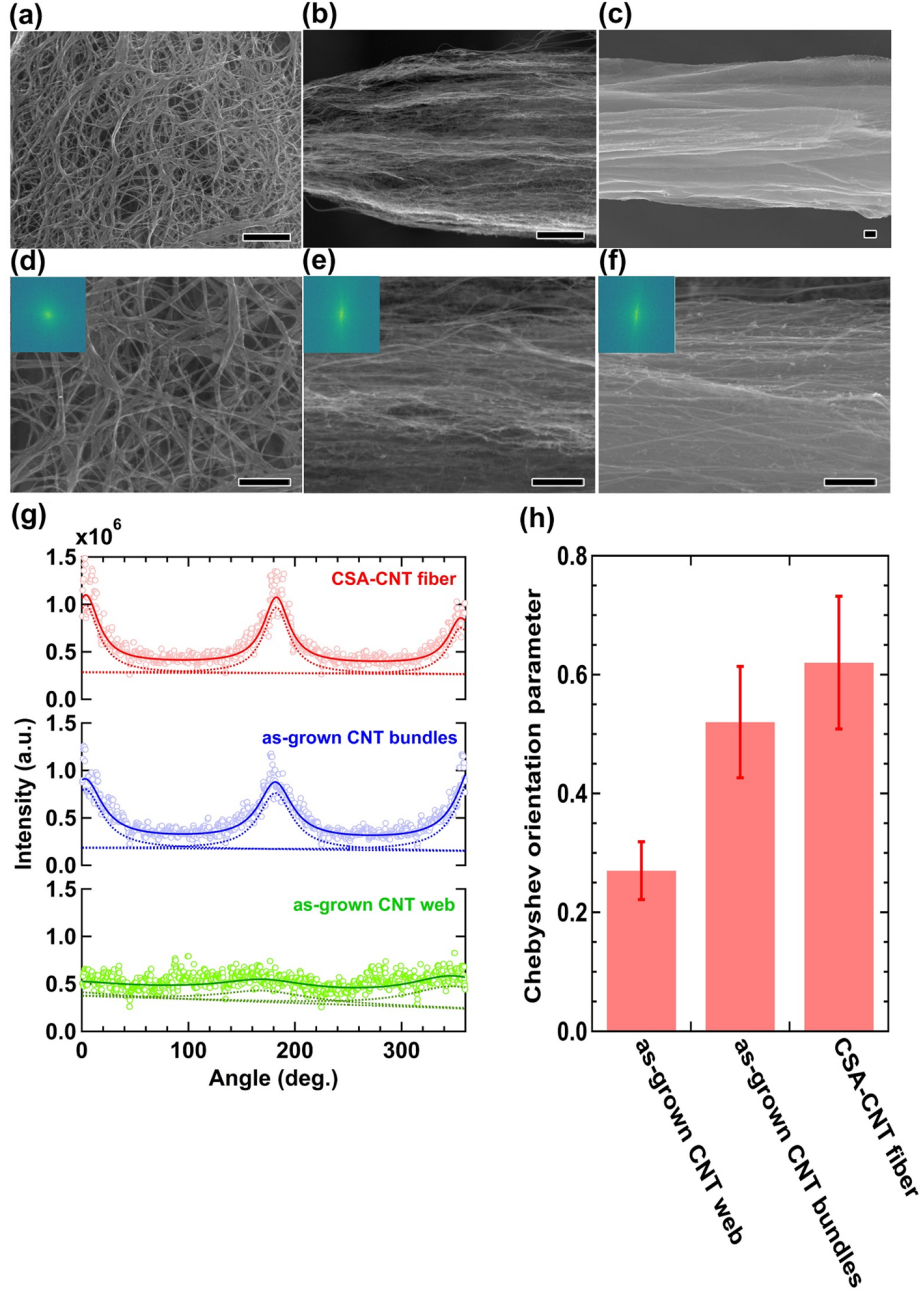
SEM images of (**a**,**d**) an as-grown CNT web, (**b**,**e**) an as-grown CNT bundles, and (**c**,**f**) a CSA-CNT fiber. Insets in (**d**–**f**) show the corresponding power spectra. Scale bar in (**a**–**c**): 1 μm. Scale bar in (**d–f**): 500 nm. (**g**) Angular intensity distributions of the CNT samples, obtained from the power spectra shown in (**d**–**f**). Fitting lines with the Lorentzian function and individual fits are depicted by the solid lines and the dotted lines, respectively. (**h**) Chebyshev orientation parameters of the CNT samples evaluated by the FFT image analysis.

Fast Fourier transform (FFT) image analysis provides information about the macroscopic alignment of CNT samples^29,37–40^. To verify how the gas rectifier affects the alignment of individual CNT bundles on the fiber surface, we compared and analyzed SEM images of as-grown CNT web produced by conventional FCCVD without using a gas rectifier, and as-grown CNT bundles (Fig. 4). Figure 4a,d show SEM images of as-grown CNT web, indicating the randomly oriented structure of the CNTs. The power spectrum obtained by FFT image analysis for as-grown CNT web exhibits a concentric pattern (inset in Fig. 4d), resulting in the obscured feature of the angular intensity distribution (Fig. 4g). This result indicates the disordered state of the CNTs, because the angular intensity distribution represents the angular distribution of CNTs. However, we found that as-grown CNT bundles produced by the modified FCCVD process consist of CNTs aligned along the fiber axis (Fig. 4b,e). The aligned structure observed in the SEM images is confirmed by a streak line in the power spectrum (inset in Fig. 4e) and a distinct peak in the angular intensity distribution (Fig. 4g). Figure 4h shows the Chebyshev orientation parameters evaluated by the FFT image analysis. As-grown CNT bundles exhibit a nearly twofold increase of the Chebyshev orientation parameter compared to that of as-grown CNT web (as-grown CNT web: 0.27 ± 0.05, as-grown CNT fiber: 0.52 ± 0.09). This result further supports the aligned structure of as-grown CNT bundles.

**Figure 5:**
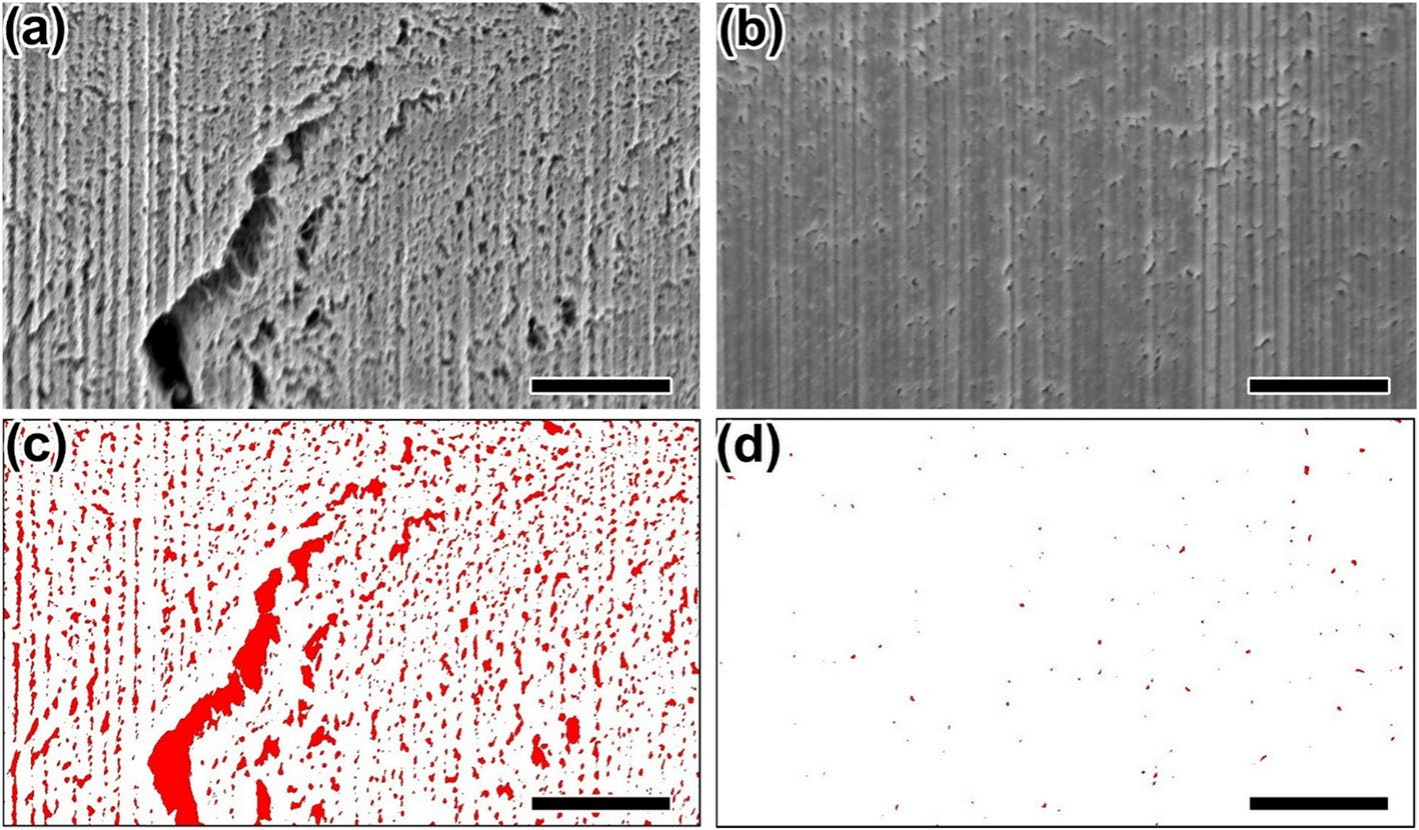
SEM images of the cross-section of (**a**) as-grown CNT bundles and (**b**) CSA-CNT fiber. Binarized images of (**c**) as-grown CNT bundles and (**d**) CSA-CNT fiber. The red areas correspond to inter-tube voids of the fibers. Scale bar: 1 μm. The binarized images were drawn by the ImageJ software (https://imagej.nih.gov/ij/).

Although the CNTs are aligned in the axial direction of as-grown CNT bundles, many voids were observed between adjacent CNTs (Fig. 4b,e). The presence of voids was also observed in the cross-sectional SEM image (Fig. 5a) and binarized SEM image (Fig. 5c) of as-grown CNT bundles, showing a porosity of 16%. To remove the voids observed in as-grown CNT bundles and further densify the fiber, as-grown CNT bundles were processed by a wet-stretching method using CSA^28^. The resulting CSA-treated, wet-stretched CNT fibers (denoted as “CSA-CNT fiber”) exhibit fewer void spaces (Fig. 4c,f) and the porosity is reduced to 0.3% (Fig. 5b,d), while maintaining the aligned structure of the CNTs (Fig. 4g) and a lower defect density (Supplementary Fig. S10). In addition, the Chebyshev orientation parameter of CSA-CNT fibers is higher than that of as-grown CNT bundles, indicating that the macroscopic alignment of CNTs is further improved by the wet-stretching process.

**Figure 6:**
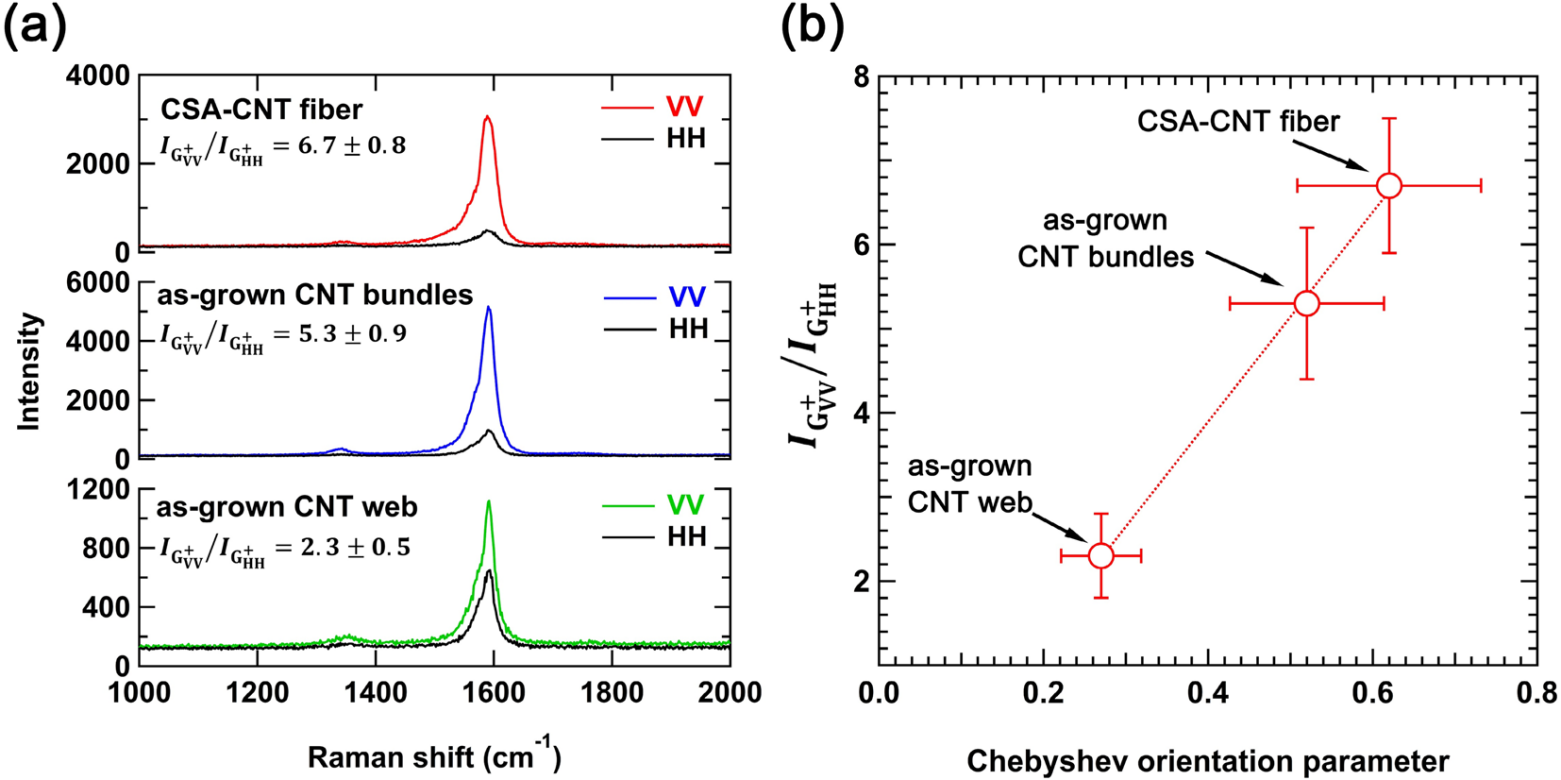
(**a**) Polarized Raman spectra of as-grown CNT web, as-grown CNT bundles, and CSA-CNT fiber, obtained with the VV and HH configurations. (**b**) Relationship between ${I}_{\tt{G}_{VV}^{+}}/{I}_{\tt{G}_{HH}^{+}}$ values and the Chebyshev orientation parameters of the CNT samples.

Polarized Raman analysis is a powerful tool for quantitative evaluation of the microscopic alignment of CNTs. It is known that the strongest G^+^ peak intensity is observed when the polarization of incident light coincides with the long axis of an individual CNT and that the signal is highly suppressed for cross-polarized light^41^. This trend is also observed in aligned CNT fibers^42^. Fig. 6a shows the polarized Raman spectra of the CNT samples, measured with the VV configuration (polarization of the incident light parallel to the fiber axis) and HH configuration (polarization perpendicular to the fiber axis). It is known that the intensity ratio of ${I}_{\tt{G}_{VV}^{+}}/{I}_{\tt{G}_{HH}^{+}}$ is correlated with the degree of alignment of CNTs^17^. This correlation is confirmed by the positive relationship between the ${I}_{\tt{G}_{VV}^{+}}/{I}_{\tt{G}_{HH}^{+}}$ values and the Chebyshev orientation parameters evaluated from the FFT image analysis (Fig. 6b). As-grown CNT bundles show a higher ${I}_{\tt{G}_{VV}^{+}}/{I}_{\tt{G}_{HH}^{+}}$ value (5.3 ± 0.9) than as-grown CNT web (2.3 ± 0.5), which supports the gas-phase alignment of CNTs inside the narrow channels of the gas rectifier. The ${I}_{\tt{G}_{VV}^{+}}/{I}_{\tt{G}_{HH}^{+}}$ value of as-grown CNT bundles is further increased by the wet-stretching process (${I}_{\tt{G}_{VV}^{+}}/{I}_{\tt{G}_{HH}^{+}}$ = 6.7 ± 0.8 for CSA-CNT fibers), indicating that the alignment of the CNTs is further improved by the wet-stretching process.

**Figure 7:**
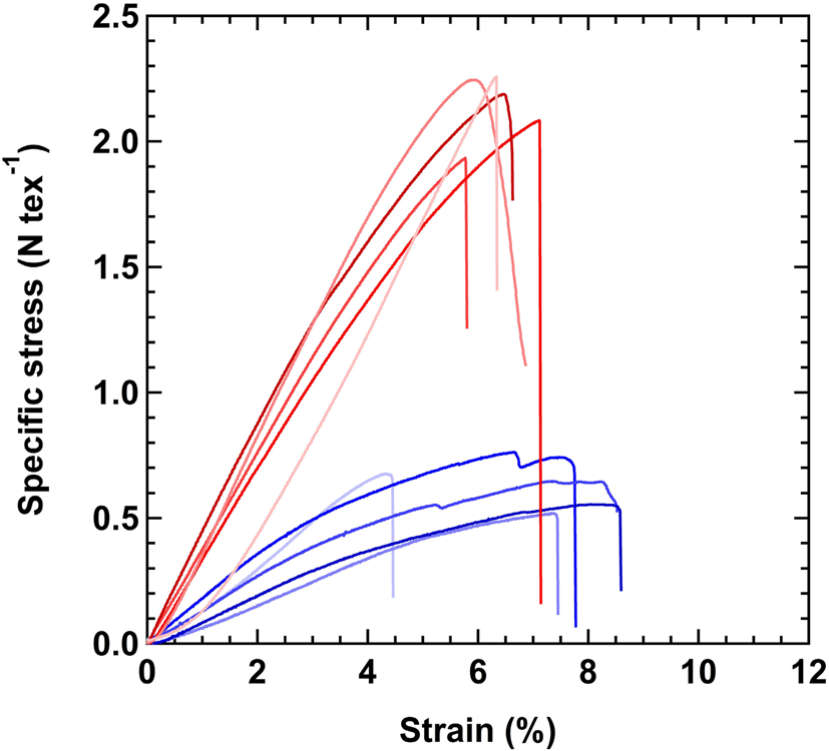
Stress–strain curves of as-made CNT fiber (blue curves) and CSA-CNT fiber (red curves).

Figure 7 shows stress–strain curves of as-made CNT fibers and CSA-CNT fibers. The linear densities of as-made CNT fibers and CSA-CNT fibers are 0.64 ± 0.08 and 0.82 ± 0.11 tex (tex = g km^−1^), respectively. As-made CNT fibers exhibit the specific tensile strength of 0.62 ± 0.09 N tex^−1^ with a Young’s modulus of 13 ± 4 N tex^−1^, and a breaking strain of 7.1 ± 1.6%. Compared to the mechanical properties of as-made CNT fibers, both the specific tensile strength and Young’s modulus of CSA-CNT fibers increase to 2.1 ± 0.1 N tex^−1^ and 39 ± 4 N tex^−1^, respectively. However, the elongation slightly decreases to 6.3 ± 0.6% for CSA-CNT fibers. We anticipate that the reduced porosity in association with the improved alignment of CNTs leads to improving the interface shear force, resulting in higher Young’s modulus and lower elongation properties of CSA-CNT fibers.

## Conclusion

We have demonstrated the one-step fabrication of aligned high-quality CNT bundles with controlled fluid properties using a gas rectifier. The advantage of our FCCVD method is the simultaneous alignment and self-assembly of floating CNTs into macroscopic CNT bundles inside a gas rectifier. The one-step formation of aligned and high-quality CNT bundles is the result of the combined effect of the gas rectification and the enhanced shear stress induced by the narrow channels of the gas rectifier. Although as-grown CNT bundles inherently possess inter-tube voids, the porosity can be significantly reduced by wet-stretching using CSA. The reduced porosity and aligned structure improve the mechanical properties of the CNT fibers. Our method is in stark contrast to the conventional fiberization techniques of CNTs, which typically use entangled CNTs as the starting material. Pre-processing with controlled fluid properties using a gas rectifier will be a key synthesis route for suppressing unnecessary entanglement in as-produced CNT samples, leading to the fabrication of high-performance CNT fibers.

## Supplementary Information

Supplementary Information.

Supplementary Video 1.
