# Supplementary material for: One step fabrication of aligned carbon nanotubes using gas rectifier
Source: Sci Rep. 2022 Jan 25;12:1285. doi: 10.1038/s41598-022-05297-6 (PMC8789815; doi:10.1038/s41598-022-05297-6)
Supplement: Supplementary file 1 — Supplementary Information. [file 41598_2022_5297_MOESM1_ESM.pdf]

Supplementary Information for

One step fabrication of aligned carbon nanotubes using gas rectifier

Toshihiko Fujimori,[1,2,*] Daiji Yamashita,[1] Yoshiya Kishibe,[2] Momoko Sakai,[2] Hirotaka Inoue,[1] Takamasa Onoki,[1] Jun Otsuka,[1] Daisuke Tanioka,[1] Takeshi Hikata,[1] Soichiro Okubo,[1] Keishi Akada,[2] Jun-ichi Fujita[2,*]

[1] Sumitomo Electric Industries, Ltd. 1-1-3, Shimaya, Konohana-ku, Osaka 554-0024, Japan

[2] Institute of Applied Physics, University of Tsukuba, Tsukuba 305-8573, Japan

*Correspondence to: fujimori-toshihiko@sei.co.jp and fujita.junichi.fu@u.tsukuba.ac.jp

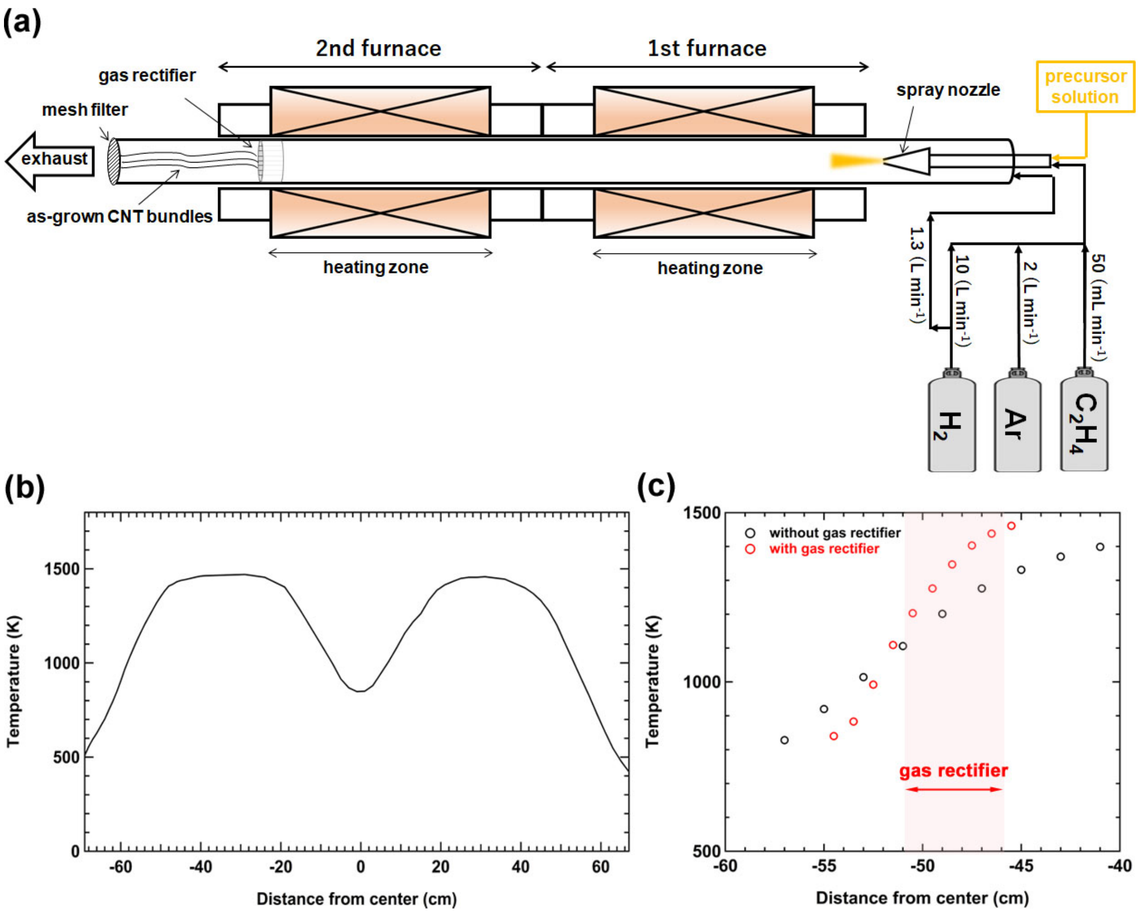

Figure S1. (a) A schematic of the horizontal reactor used in this study. (b) Temperature profile along the horizontal direction of the reactor (set temperature of the furnace: 1473 K). Due to the heat transfer through thermal radiation of the heater, temperature of the spray nozzle is kept at 383-423 K during the synthesis of CNTs. Since there are no heating zone between first and second furnaces, a temperature drop is observed at the center of the reactor. (c) Temperature profiles at the exit of the reactor, obtained with (red open circle) and without (black open circle) using the gas rectifier. The difference in the temperature profiles measured with and without the gas rectifier likely originates from the suppression of heat transfer by the gas rectifier.

Table S1. Experimental condition of the feedstock.

Molar flow rate			Atomic ratio		
Fe (mol min^{-1})	C (mol min^{-1})	S (mol min^{-1})	Fe:C	S:C	Fe:S
1.9×10^{-5}	1.1×10^{-2}	2.1×10^{-5}	0.0017	0.0019	0.90

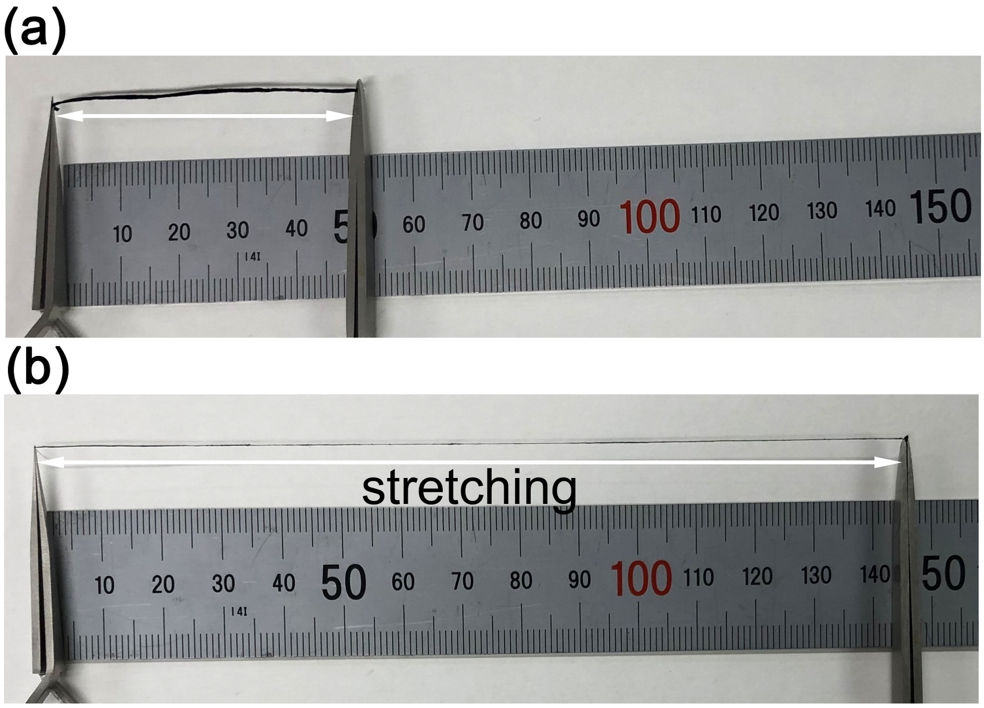

Figure S2. Photos of a thick tread of as-grown CNT bundles (**a**) before and (**b**) after the wet-stretching process using CSA.

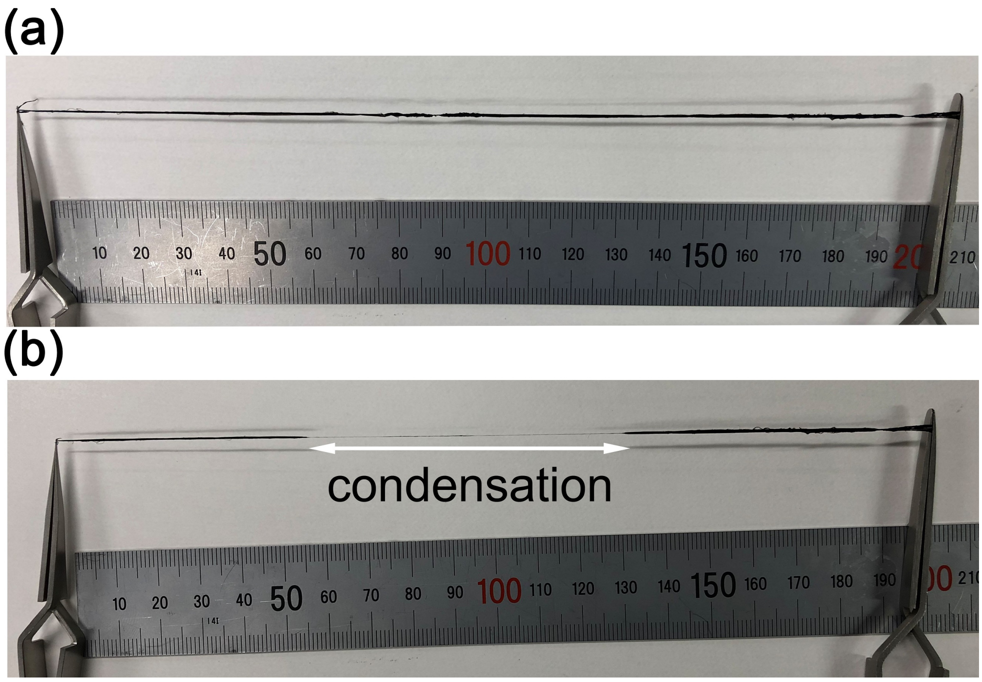

Figure S3. Photos of a thick thread of as-grown CNT bundles (**a**) before and (**b**) after the liquid condensation using chloroform.

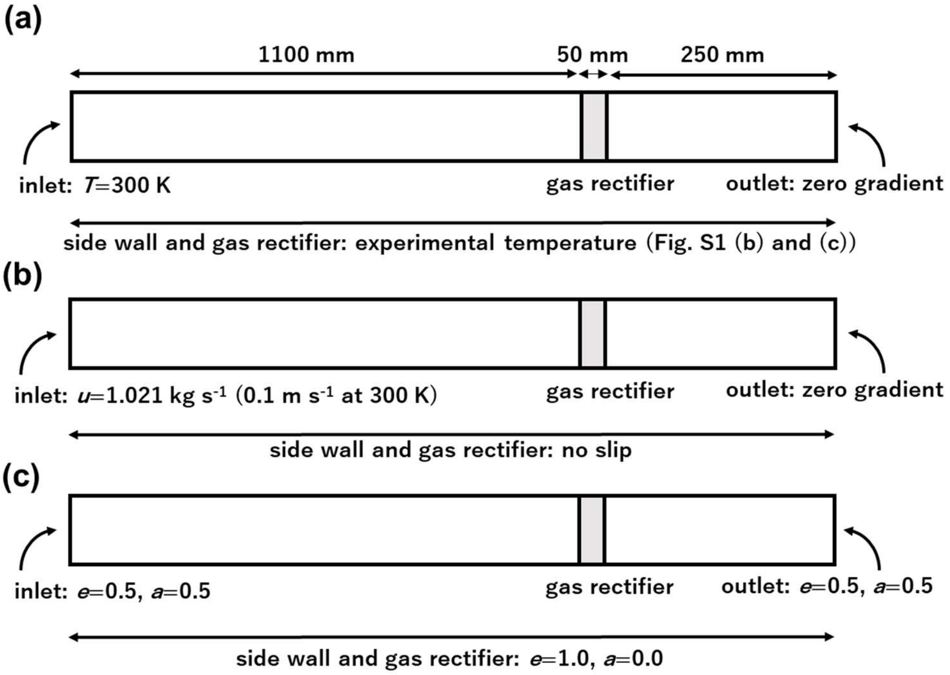

Figure S4. Boundary conditions for (a) temperature, (b) velocity, and (c) radiation in the CFD simulation. *T*, *u*, *e*, and *a* indicate temperature, velocity, emission coefficient, and absorption coefficient, respectively.

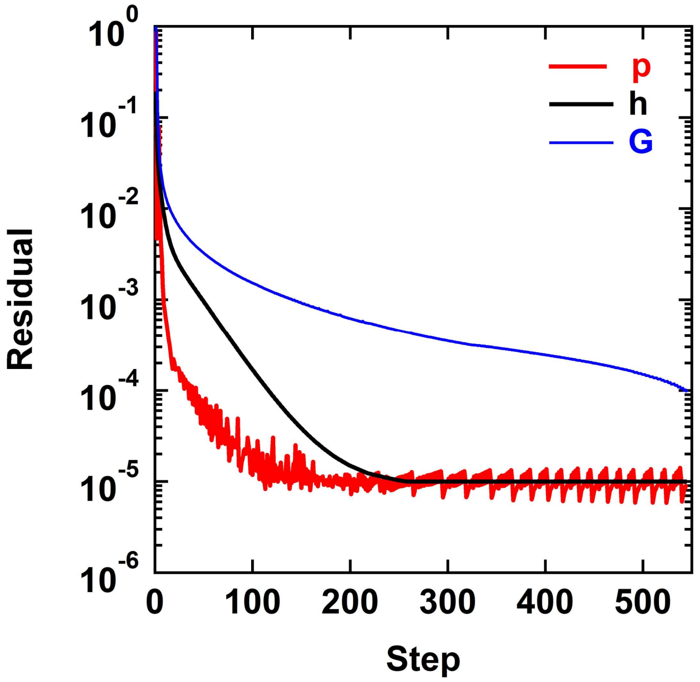

Figure S5. Convergence processes of the residual pressure (*p*), heat (*h*), and incident radiation intensity (*G*) in the CFD simulation. In the final step, the residuals reached to the convergence criteria (<10^{-4}).

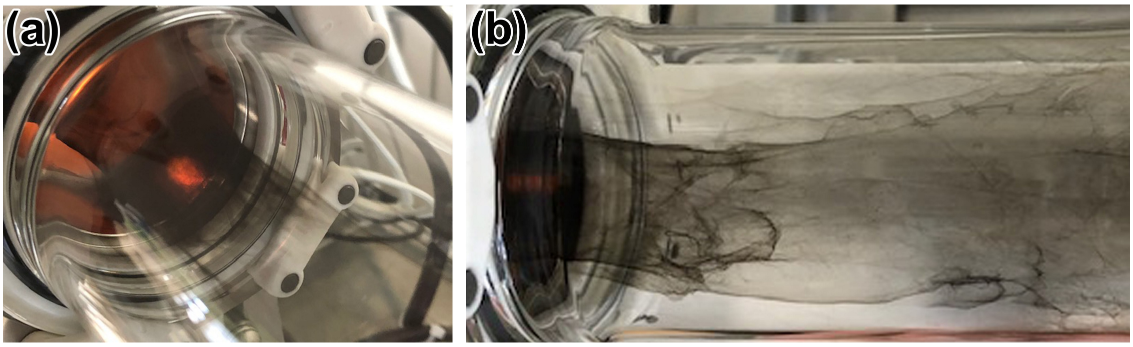

Figure S6. Photos of a hollow aggregate of CNTs synthesized without using a gas rectifier. (a) A hollow aggregate of CNTs at the exit of the reactor and (b) that migrating along the direction of gas flow (from left to right in the photos).

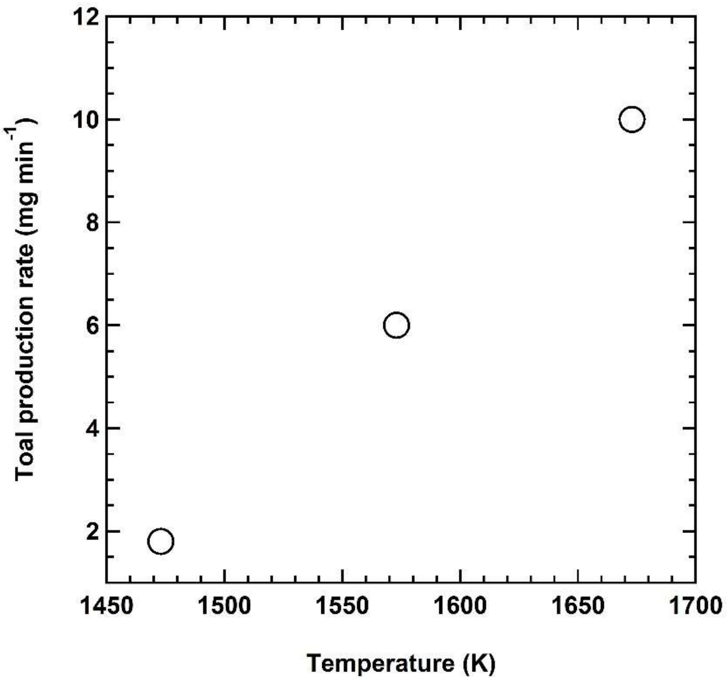

Figure S7. Total production rate of the CNT samples as a function of synthesis temperature.

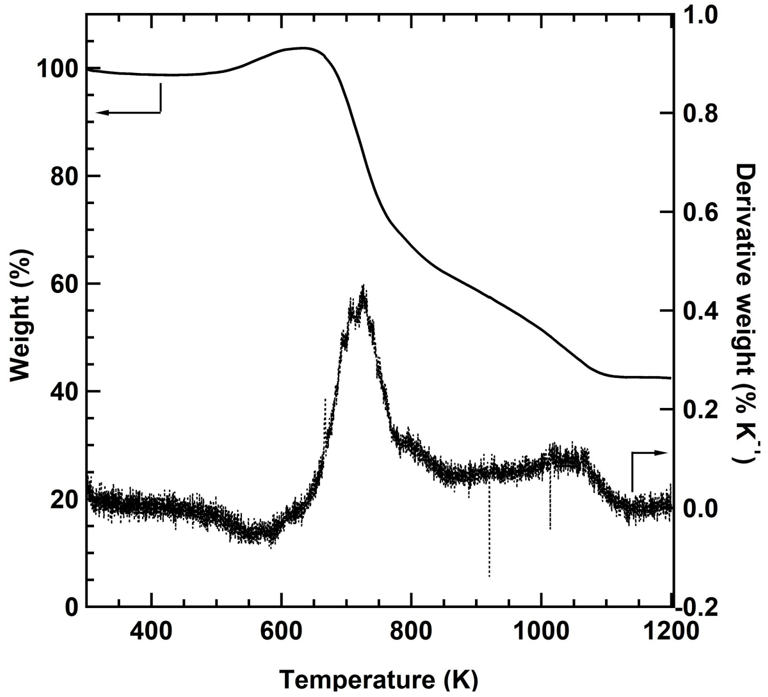

Figure S8. TG curves of as-grown CNT bundles synthesized at 1673 K. Residual amount at 1200 K corresponds to the catalyst residue (particles of iron oxides), indicating that the residual amount is 43 wt% for as-grown CNT bundles. Increase of the weight at 500-650 K originates from the oxidation of iron particles.

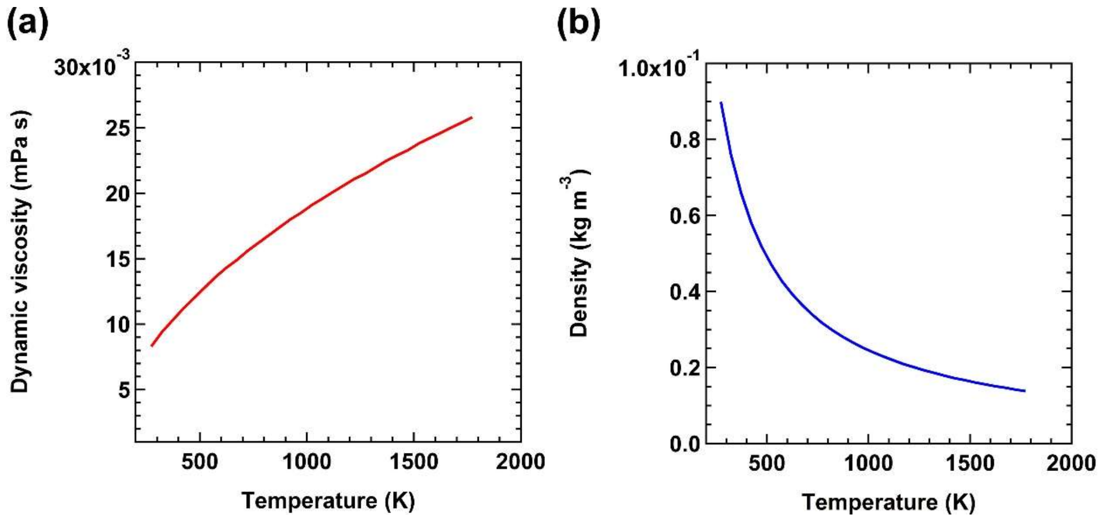

Figure S9. (a) Dynamic viscosity $\mu(T)$ of hydrogen gas as a function of temperature T, estimated by Sutherland's viscosity law using the Sutherland constant, S=72 and a reference dynamic viscosity of μ(273 K)=0.0088 (mPa s).[1] (b) Calculated density, $\sigma(T)$ of hydrogen gas as a function of temperature, estimated by the equation of state for ideal gas using σ(273 K) =0.0899 (kg m^{-3}).[2]

Table S2. Parameters for calculating Reynolds numbers.

position	T (K)	$\rho(T)$ (kg m^{-3})	$u(T)$ (m s^{-1})	$\mu(T)$ (Pa s)	D_{H} (m)	Shape of cross-section	R_e
i	1460	0.0168	0.95	2.32×10^{-5}	0.045	Circle 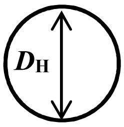 reaction tube	31
iii	1346	0.0182	1.70	2.22×10^{-5}	0.0016	Square 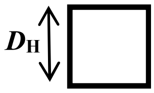 a channel of the rectifier	2.2

The Reynolds number in a pipe is expressed by

$$R_e = {}^{\rho(T)u(T)D_H}\big/_{\mu(T)} \ , \tag{S1}$$

$$D_H = {}^{4A}/_{P} \ , \tag{S2}$$

where $\rho(T)$ is the density, $u(T)$ is the characteristic z-velocity, $\mu(T)$ is the dynamic viscosity, and T is the temperature of the process gas. D_{H} is the hydraulic diameter, A is the cross-sectional area, and P is the wetted perimeter of the pipe.

The hydraulic diameter of the reaction tube is identical to the diameter of the reaction tube (D_{H}=($4\pi r^2$) / ($2\pi r$)=$2r$, where r is the radius of the tube). The hydraulic diameter of the channel of the gas rectifier corresponds to the length of one side of the square-shaped pore (D_{H}=($4L^2$) / ($4L$)=L, where L is the length of one side of the square-shaped pore).

The Reynolds number around the position **i** is calculated as

$$R_e(reaction\ \ tube) = \frac{0.0168(kg\ \ m^{-3})\times 0.95(m\ \ s^{-1})\times 0.045(m)}{2.32\times 10^{-5}\ \ (Pa\ \ s)} = 31.$$

And the Reynolds number at position **iii** is calculated as

$$R_e(channel) = \frac{0.0182(kg\ \ m^{-3})\times 1.70(m\ \ s^{-1})\times 0.0016(m)}{2.22\times 10^{-5}\ \ (Pa\ \ s)} = 2.2.$$

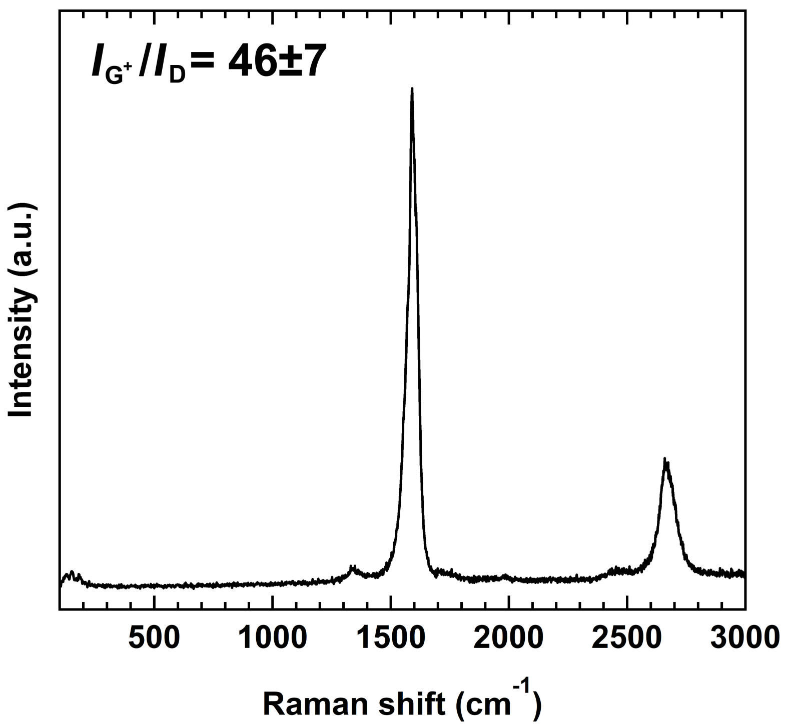

Figure S10. A representative Raman spectrum of CSA-CNT fiber, measured with the 532 nm laser excitation. Considered the experimental error, there is a negligible change in the I_{G^+}/I_D values between CSA-CNT fiber (I_{G^+}/I_D=46±7) and as-grown CNT bundles (I_{G^+}/I_D=50±7).

Supplementary References

1. Kaye G. W. and Laby, T. H. *Table of Physical and Chemical Constants and Some Mathematical Functions* (Longman Publishing Group, London, 1986).
2. National Astronomical Observatory of Japan (Ed.). *Chronological Scientific Tables 2001*, pp. 450 (Maruzen Publishing Co., Ltd., Tokyo, 2001).
